# Developmental profiling of microRNAs in the human embryonic inner ear

**DOI:** 10.1371/journal.pone.0191452

**Published:** 2018-01-26

**Authors:** Duncan M. Chadly, Jennifer Best, Cong Ran, Małgorzata Bruska, Witold Woźniak, Bartosz Kempisty, Mark Schwartz, Bonnie LaFleur, B. J. Kerns, John A. Kessler, Akihiro J. Matsuoka

**Affiliations:** 1 Department of Otolaryngology and Head and Neck Surgery, Feinberg School of Medicine, Northwestern University, Chicago, Illinois, United States of America; 2 Department of Anatomy, Poznań University, Poznań, Poland; 3 HTG Molecular Diagnostics, Inc., Tucson, Arizona, United States of America; 4 Department of Neurology, Feinberg School of Medicine, Northwestern University, Chicago, Illinois, United States of America; 5 Department of Communication Sciences and Disorders, Northwestern University, Evanston, Illinois, United States of America; 6 Hugh Knowles Center for Hearing Research, Northwestern University, Evanston, Illinois, United States of America; University of Massachusetts Medical School, UNITED STATES

## Abstract

Due to the extreme inaccessibility of fetal human inner ear tissue, defining of the microRNAs (miRNAs) that regulate development of the inner ear has relied on animal tissue. In the present study, we performed the first miRNA sequencing of otic precursors in human specimens. Using HTG miRNA Whole Transcriptome assays, we examined miRNA expression in the cochleovestibular ganglion (CVG), neural crest (NC), and otic vesicle (OV) from paraffin embedded (FFPE) human specimens in the Carnegie developmental stages 13–15. We found that in human embryonic tissues, there are different patterns of miRNA expression in the CVG, NC and OV. In particular, members of the miR-183 family (miR-96, miR-182, and miR-183) are differentially expressed in the CVG compared to NC and OV at Carnegie developmental stage 13. We further identified transcription factors that are differentially targeted in the CVG compared to the other tissues from stages 13–15, and we performed gene set enrichment analyses to determine differentially regulated pathways that are relevant to CVG development in humans. These findings not only provide insight into the mechanisms governing the development of the human inner ear, but also identify potential signaling pathways for promoting regeneration of the spiral ganglion and other components of the inner ear.

## Introduction

MicroRNAs (miRNA) are a class of endogenously expressed small non-coding RNAs that function in RNA silencing and post-transcriptional regulation of gene expression. The human genome encodes more than 1000 miRNAs that may target as many as 60% of human protein-encoding genes [1]. miRNAs regulate stem/progenitor cell proliferation and differentiation as well as organ development and function [1,2], and more specifically they have been shown to be involved in mouse inner ear development and maturation [3,4]. Disruption of the production of miRNAs causes profound inner ear malformation and deafness [5–8], and a mutation in a single miRNA, miR-96, causes deafness in both humans and mice [9,10]. In addition, the miR-183 family (miR-96, miR-182, and miR-183) is differentially expressed in the mouse inner ear as compared to other organs [3,11]. However, little is known about miRNA expression in human inner ear development.

The HTG EdgeSeq System (HTG Molecular Diagnostics, Tucson, AZ, USA) is an automated miRNA expression analysis platform that can deliver reliable results on a previously generated histopathology slide (i.e., a formalin-fixed paraffin-embedded (FFPE) slide) [12–15]. This technique is used here to define the miRNA expression profile in otic precursors in human FFPE specimens at Carnegie developmental stages 13–15 (corresponding to 32 to 35 postovulatory days) [16,17]. We demonstrated in human embryonic tissues that members of the miR-183 family are differentially expressed in the cochleovestibular ganglion (CVG) at embryonic stage 13 in the human inner ear as compared to nearby neural crest (NC) and otic vesicle (OV) tissues. We further identified transcription factors that are differentially targeted in the CVG compared to the other tissues from stages 13–15, and performed gene set enrichment analyses to determine differentially regulated signaling pathways, many of which are relevant to CVG development in humans.

## Materials and methods

### Sample collection

**Fig 1:**
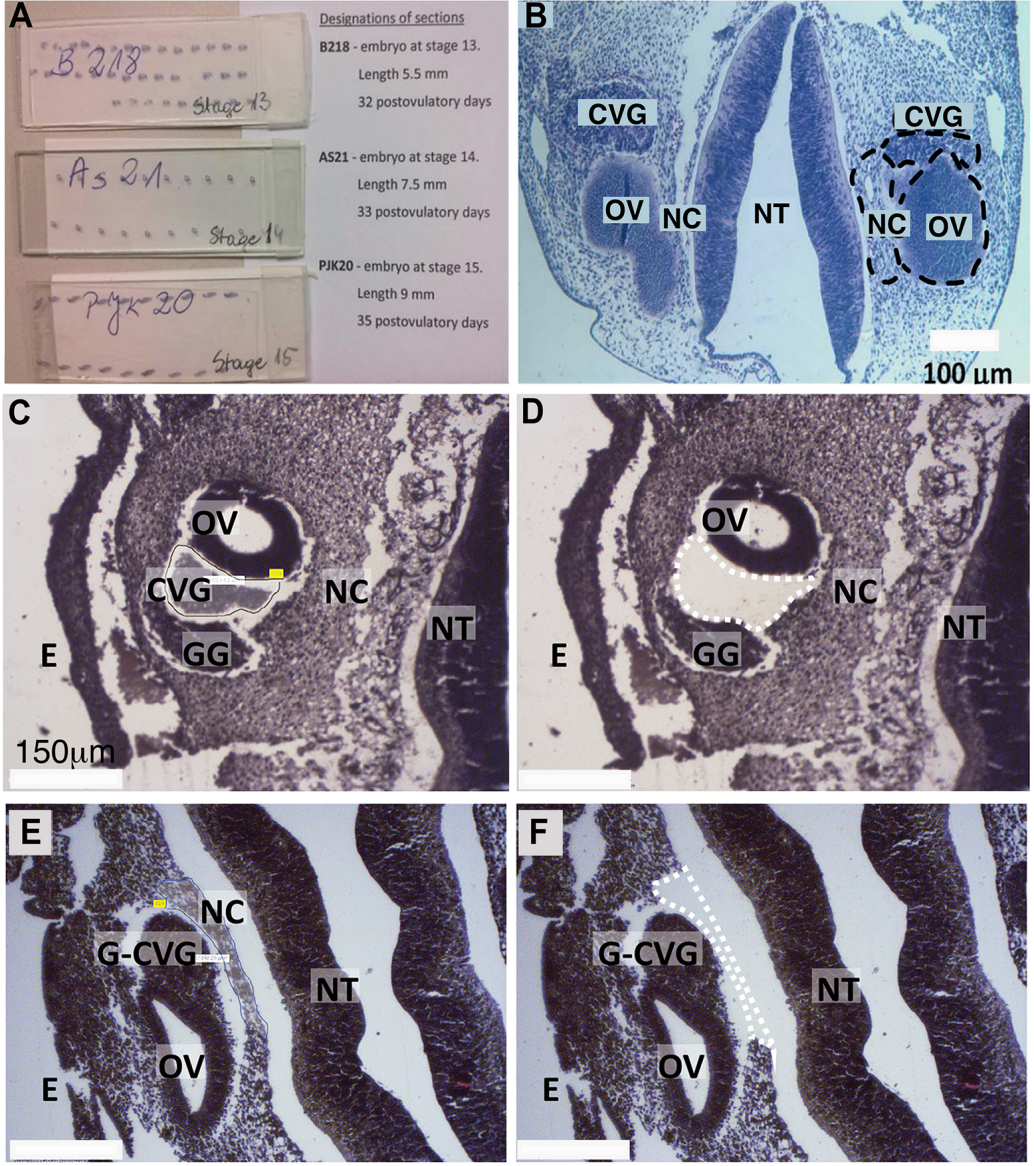
Laser-microdissection of FFPE-human inner ear slides. (A) FFPE slides containing embryonic tissues. (B) Coronal section of a fetus with highlighted regions of interest (dashed black circles, CVG, NC, and OV). Further images are provided showing tissue before (C, E) and after (D, F) laser-captured microdissection of CVG (dashed white lines in D) and NC tissues (dashed white line in F). Other developmental regions also are highlighted. CVG: cochlear-vestibular ganglions, OV: otic vesicle, NC: neural crest; GG: geniculate ganglion, G-CVG: geniculate-cochleovestibular ganglions, E: epithelium; and NT: neural tube. Scale bar = 100 μm (B) and 150 μm (C-F).

Three FFPE human embryo specimens were provided by Dr. Malgorzata Bruska in the Department of Anatomy, Poznań University, Poland. These slides had been used previously for educational purpose at Poznań University. The slides were labeled (Fig 1A) as follows: 1) B218—embryo at the Carnegie stage 13, 32 post ovulatory days; 2) AS21—embryo at the Carnegie stage 14, 33 post ovulatory days; 3) PJK20—embryo at the Carnegie stage 15, 35 post ovulatory days. Carnegie developmental stages 13, 14, and 15 in human development correspond to embryonic days 11 (E11), E11.5, and E12, respectively in mouse. Each FFPE slide was placed in a small Coplin jar in fresh xylene to remove the cover slip. The xylene solution was changed every 72 hours. Each cover slip was removed from the slide within 1 week without difficulties. We chose three areas of interest, the CVG, NC, and OV, because we are interested in miRNA expression during CVG development as compared to NC or OV tissues. Note that CVG consists of both the spiral (auditory) and vestibular ganglions and thus includes the developmental precursors of spiral ganglion neurons (SGNs) [18]. The three areas were dissected using a Zeiss Palm laser catapulting micro-dissection system and collected in CZMI Adhesive Cap 500 collection tubes (Carl Zeiss, Oberkochen, Germany). An example of the dissection of the CVG and NC area is provided in Fig 1B–1F. Samples were provided as formalin-fixed, paraffin-embedded, hematoxylin and eosin stained tissue. Profiles were collected for three tissue types (CVG, NC, and OV) at three developmental time points (Carnegie developmental stage 13, 14, and 15, corresponding to 33, 34, and 35 postovulatory days) with three technical replicates. Each Adhesive Cap collection tube contained approximately 5 cross-sectional slices of the area of interest. The samples were labeled as described in S1 Table.

### HTG Edgeseq miRNA whole transcriptome assay (WTA)

HTG Edgeseq miRNA whole transcriptome assay (WTA) is a next-generation sequencing (NGS) application that measures the expression of 2,255 human miRNAs described in the miRBase v20 database without the need for extracting RNA [19]. Lysis of collected cells in each tube was performed by applying 55μL of HTG Lysis Buffer (HTG Molecular, Tucson, AZ) to the cap and incubating upside down for 30 minutes at room temperature. After 30 minutes the sample was spun down and 2.8μL of Proteinase K (HTG Molecular, Tucson, AZ, USA) was added, and the sample was then incubated for 180 minutes at 50°C. From each prepared sample, 25μL were added per well to a 96-well sample plate. Human fetal Brain RNA was added to one well at 25 ng/well to serve as a process control. Samples were run on an HTG EdgeSeq Processor using the HTG EdgeSeq miRNA WTA (HTG Molecular, Tucson, AZ, USA). This assay revolves around nuclease protection, where a pre-selected miRNA population is protected with proprietary protection probes, followed by degradation of all nonhybridized probes and non-targeted RNA by S1 nuclease. This step results in a 1:1 stoichiometric ratio of probes to targeted RNA. Following the processor step, samples were individually barcoded (using a 16-cycle PCR reaction to add adapters and molecular barcodes). Barcoded samples were individually purified using AMPure XP beads (Beckman Coulter, Brea, CA, USA) and quantitated using a KAPA Library Quantification kit (KAPA Biosystem, Wilmington, MA, USA). The library was sequenced on an Illumina MiSeq (Illumina, Inc., San Diego, CA) using a V3 150-cycle kit with two index reads. PhiX was spiked into the library at 5%; this spike-in control is standard for Illumina sequencing libraries. Data were returned from the sequencer in the form of demultiplexed FASTQ files, with one file per original well of the assay. The HTG EdgeSeq Parser (HTG Molecular, Tucson, AZ, USA) was used to align the FASTQ files to the probe list to collate the data. Data were provided as data tables of raw, quality control (QC) raw, counts per million (CPM), and median normalized counts.

### Quality control

Baseline performance characteristics were established using Human Universal Reference RNA (uRNA) (Agilent genomics, Santa Clara, CA, USA) across all 96 wells on three sequencing runs of 96-well, with each plate processed on a different HTG EdgeSeq processor. Log_2_ counts per million (log_2_(CPM)) standardization was used to transformed counts and adjusted for total reads within a sample [20]. The RNA-sequence data consist of a matrix of read count *r*_*gi*_, for RNA samples *i* = 1 to *n*, and genes *g* = 1 to *G*. *Ri*, the total number of mapped reads for sample *I*, is defined as:

$$Ri=\sum_{g=1}^{G}r_{gi}$$

The log_2_(CPM) values for each probe within a sample was defined as:

$$y_{gi}=\log_{2}\left(\frac{r_{gi}+0.5}{R_{i}+1.0}\times 10^{6}\right)$$

Where *y*_*gi*_ is normalized count for sample *i* and gene *g*. The counts are offset by 0.5 to avoid taking the log of zero, and total counts are offset by 1 to ensure the ratio is strictly less than 1 [20].

Baseline performance was established on the 96 technical replicates after normalization. Statistical process control methods were used to establish this expected performance where averaged ANT values (negative control) were calculated for each sample; a grand mean was calculated by taking the average of these averaged ANT values; the difference between each averaged ANT value and the grand mean, Δmean (averaged sample mean–grand mean) and standard deviation (SD) of the Δmean was calculated was for each sample. Acceptable Δmean values are those within ± 2SD average ANT. The graphical representation of the statistical process control, the quality control chart, for ANT using 96 miRNA technical replicates is shown in S1 Fig.

### miRNA differential expression analysis and principal components analysis

A median normalization was used to transform and standardize data before data analyses [21]. The primary statistical analysis was performed within the three time points and examined the differences in the pattern of expression among CVG, NC, and OV cell types. The R Bioconductor package (version 3.5) Linear Models for Microarray and RNA-Seq Data (*limma*) was used for the statistical analyses [22]. Secondary statistical analysis examined differences in the pattern of expression over time within the CVG, NC, and OV cell types. The R Bioconductor package (version 3.5) time course was used for this analysis [23]. The top 100 differentially expressed miRNA molecules across time points, ranked by significance (Hotelling’s t-squared statistic), were determined for each of the three tissues. The maximal test statistic for the comparisons across three time points (stage 13 vs. 14, 14 vs. 15, and 13 vs. 15) was tabulated; differentially expressed miRNAs, indicating potential developmental relevance, were then identified by comparing these lists. Differential expression analyses included a correction for false discovery rate (FDR) was performed using the Benjamini-Hochberg Method [24]. The R Bioconductor (version 3.5) was used for the analysis [25]. To understand the primary determinant of miRNA expression and to examine sample clustering, Principal Component Analysis (PCA) was performed to identify the drivers of differences. Normalized miRNA expression values were assembled into a matrix with rows of different sample types and columns of miRNAs.

### Over-connected gene analysis

Hypergeometric enrichment was used to identify genes significantly over-connected to miRNAs detected in our differential expression datasets. Gene targets of miRNA were predicted by combining the TargetScan (version 7.1) nonconserved and conserved family databases, using all predicted targets for the human species [26]. This provided targets for 1408 of the miRNA families that were used for over-connected gene analysis. A list was compiled of all miRNA-gene links, allowing for multiple targets per miRNA. The hypergeometric distribution was used to compute *p*-values:

$$p=\frac{\left(\begin{array}{c} K\\ k \end{array})(\begin{array}{c} N-K\\ n-k \end{array}\right)}{\left(\begin{array}{c} N\\ n \end{array}\right)}$$

where *p* is the probability that a gene would be targeted at least *k* times from a finite population under sampling without replacement if each gene had an equal probability of being targeted, *N* is the total number of miRNA-gene objects for all miRNA tested in the differential expression experiment, *K* is the number of times a given gene is represented in *N*, *n* is the number of miRNA-genes with parent miRNA that are considered differentially expressed (*p* < 0.05), and *k* is the number of miRNA-gene objects for a given gene represented by differentially expressed miRNA. This analysis was repeated for each miRNA differential expression dataset. The data were filtered for FDR corrected *p*-value < 0.10.

Ensemble Gene Ontology (GO) annotations were obtained using the R Bioconductor (version 3.5) BiomaRt package [27]. The complete list of over-connected genes was filtered to include only GO terms containing “transcription factor” in their annotation. To visualize these results, over-connected gene network graphics were created for several differential expression datasets using the R igraph package (version 1.0.1) [28]. Significantly enriched miRNA-gene objects are represented with miRNA and genes as nodes. Node size reflects the number of connections made to each gene. Network graphics were created for all tissue comparisons with few enough over-connected transcription factors for the network plot to remain sparse enough to be useful.

### Gene set enrichment analysis

The R Bioconductor mdgsa package (version 1.8.0) was used for pathway analysis of miRNA differential expression [29]. R (version 3.3.3 (Another Canoe), released on 03/06/2017) was additionally used for all subsequent statistical and computational analyses. The same TargetScan databases used for pathway analysis were used to predict miRNA-gene associations. Pathway-gene associations were taken from the Consensus Pathway Database (ConsensusPathDB, release 31), which combines 32 publically available interaction resources to provide information on protein interaction, signaling reactions, metabolic reactions, gene regulation, genetic interaction, drug-target interaction, and biochemical pathways [30]. See further details in S1 File.

Integrated gene set analysis was performed following the method of Garcia-Garcia et al. [31], incorporating the paradigm shift proposed by Godard and Eyall to prevent the influence of knowledge bias [32]. See details in S1 File. Following this approach, the pathway database (containing entries linking pathways to genes) was converted to an annotation database containing pathways linked to the miRNA targeting their genes using TargetScan predictions. Gene set analysis was then performed using mdgsa package, which fits a logistic regression model relating the probability of a gene belonging to the gene set with the value of the *r* statistic. The package outputs a log odds ratio for each interrogated gene set, along with raw and false discovery adjusted *p*-values [24]. A positive log odds ratio indicates more targeting of a pathway by miRNA in case 1 compared to case 2 for each case 1 vs. case 2 comparison.

Alternatively, we identified significantly altered pathways using another type of gene set enrichment analysis. This analysis was conducted on the transformed gene expression from the miRNA analysis, which generated *r* statistics for each gene using the Garcia-Garcia method [31]. The subsequent enrichment score is generated using R the Gene Set Variation Analysis (GSVA) package (version 1.24.1) on each gene set using ranking statistics similar to the Kolmogorov-Smirnov test described in Hanzelmann et al [33]. Gene Ontology Pathway Database (Broad Institute, downloaded on 08/16/2017) was the gene set database, assayed in each sample for which it included a total of 5917 gene sets that are known to be involved in biological processes, cellular components and molecular functions [34,35]. The package outputs an GSVA enrichment score for each interrogated gene set. The statistical significance is determined by *p*-values that are calculated using Baysian goodness of fit model using the R limma package (version 3.32.5).

To reduce functional redundancy within the Gene Ontology terms identified with gene set enrichment analysis with GSVA enrichment score, we next used REVIGO (http://revigo.irb.hr/), a web based program where *sim*_*Lin*_ is Lin’s sematic similarity measure [36], *S (c*_*1*_, *c*_*2*_*)* is the set of common ancestors of terms *c*_*1*_, *c*_*2*,_ and *p(c)* is the probability of a term [37,38]. The score is termed uniqueness in REVIGO. The previously calculated enrichment *p*-value for each term was not factored into the query since the aim was to reduce functionally overlapping or semantically redundant pathways; however, only significantly enriched terms were included in the query. Given that only one percent of randomly paired terms will be allowed at a uniqueness score of *C* = 0.53, a less stringent *C* = 0.7 was used to allow for a slightly semantically diverse list without significantly compromising the goal of reducing redundancy.

### Computational prediction of the miR-183 family targeted genes

To investigate how the miR-183 family targets gene expression in human inner ear development, we performed computational target prediction for the miR-183 family (miRNA-96-5p, miR-182-5p, and miR-183-5p). As miRNA can have numerous targets, we first computationally predict the targets by using a miRNA prediction algorithm, TargetScanHuman (release 7.1: June 2016). TargetScan predicts biological targets of miRNAs by searching for the presence of conserved 8mer, 7mer, and 6mer sites that match the seed region of each miRNA [39,40]. We then compared these results to the Deafness Variation Database (version 8.0), as this database provides a most comprehensive list of genetic variation in genes known to be associated with human deafness (http://deafnessvariationdatabase.org/download) [41,42]. Relevant targets were thereby identified as the intersection of TargetScan predictions and genes associated with deafness as listed in Deafness Variation Database.

## Results

### Differential miRNA expression among tissues for three developmental stages

**Fig 2:**
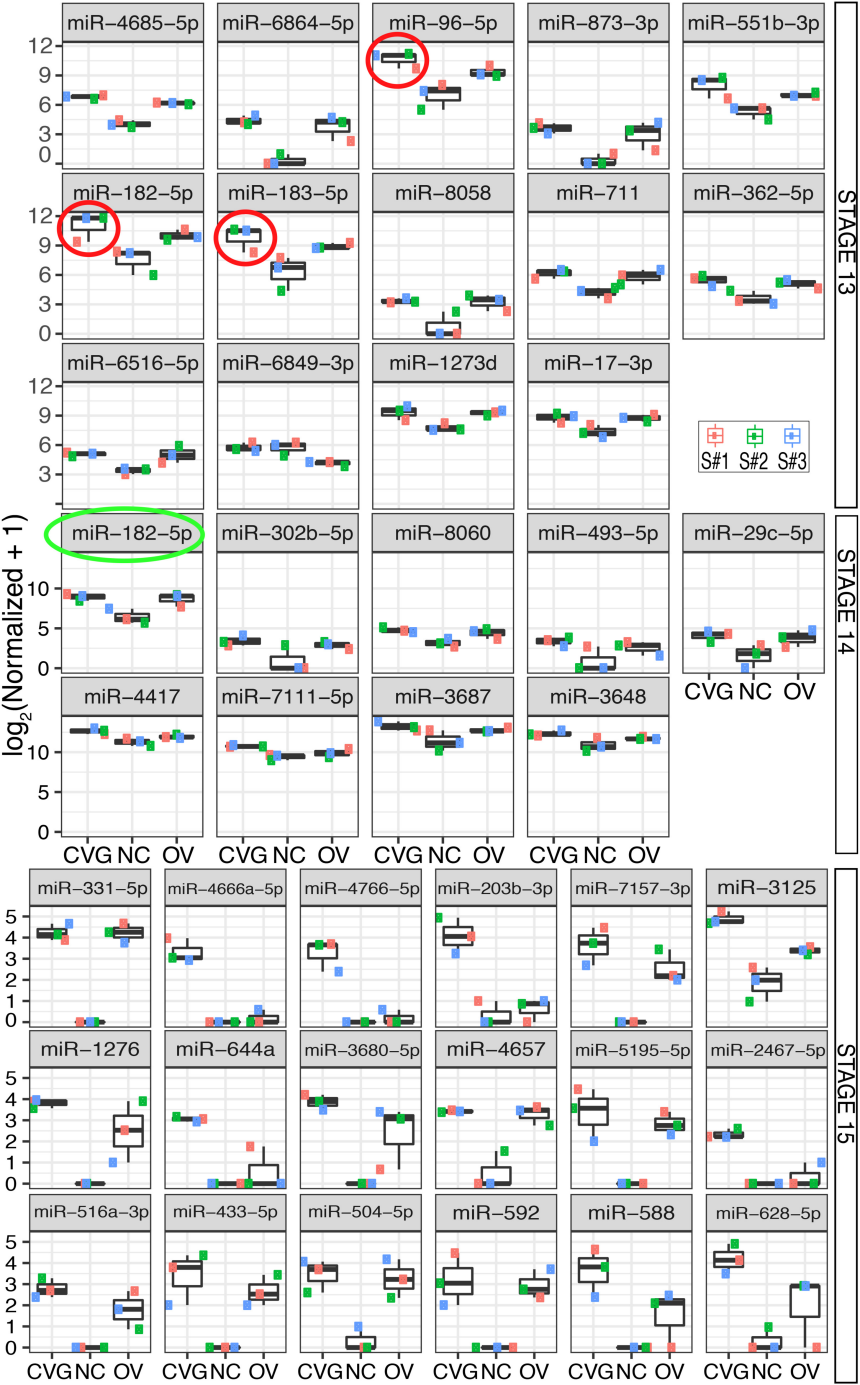
Differential expression of miRNA across tissues. Differentially expressed miRNA between three tissue types (CVG, NC, and OV) with *p* < 0.05 and false discovery adjusted *p* < 0.25, 0.50, or 0.10 (based on the smallest *p-*value out of the three possible tissue comparisons) are plotted for tissue from Carnegie stages 13, 14, and 15, respectively. MicroRNA from the miR-183 family is circled in red (stage 13) or green (stage 14).

Boxplots are shown in Fig 2 for expression of differentially expressed miRNA where CVG has the highest expression level at three different time points across the three tissue types, CVG, NC, and OV. These results show differentially expressed miRNA with *p* < 0.05 and false discovery rate (FDR) adjusted *p* < 0.25, 0.50, or 0.10 for tissue from Carnegie stages 13, 14, and 15, respectively. The smallest *p*-value out of the three possible tissue comparisons for each miRNA was considered. FDRs were controlled separately for each time point to minimize type II error while still maintaining a reasonable number of results, keeping in mind that a FDR controlled at 0.50 implies that 50% of differential expression results are expected to be false positives. Triplicate measurements were taken by collecting tissue from three different sections of one biological specimen. All three tissue samples within a time point are similarly from a single biological specimen.

Note that at the Carnegie developmental stage 13, members of the miR-183 family (miR-96, miR-182, and miR-183) were highly expressed in CVG and OV as compared with NC (for CVG vs NC comparison, *p* = 0.0009, 0.003, and 0.0045; with FDR correction, *p* = 0.27, 0.40, and 0.41, respectively). Also, note that expression of the entire miR-183 family was the highest in CVG tissue at stage 13 (see red circles on Fig 2). The trend was not seen at Stage 14, where the only member of the miR-183 family that was upregulated was miR-182 (*p* = 0.004; with FDR correction *p* = 0.27) (indicated with a green circle in Fig 2). At stage 15, no members of the miR-183 family were observed. Boxplots for expression of the top-20 differentially expressed over the three time points for CVG, NC, and OV are shown in S2 Fig.

**Fig 3:**
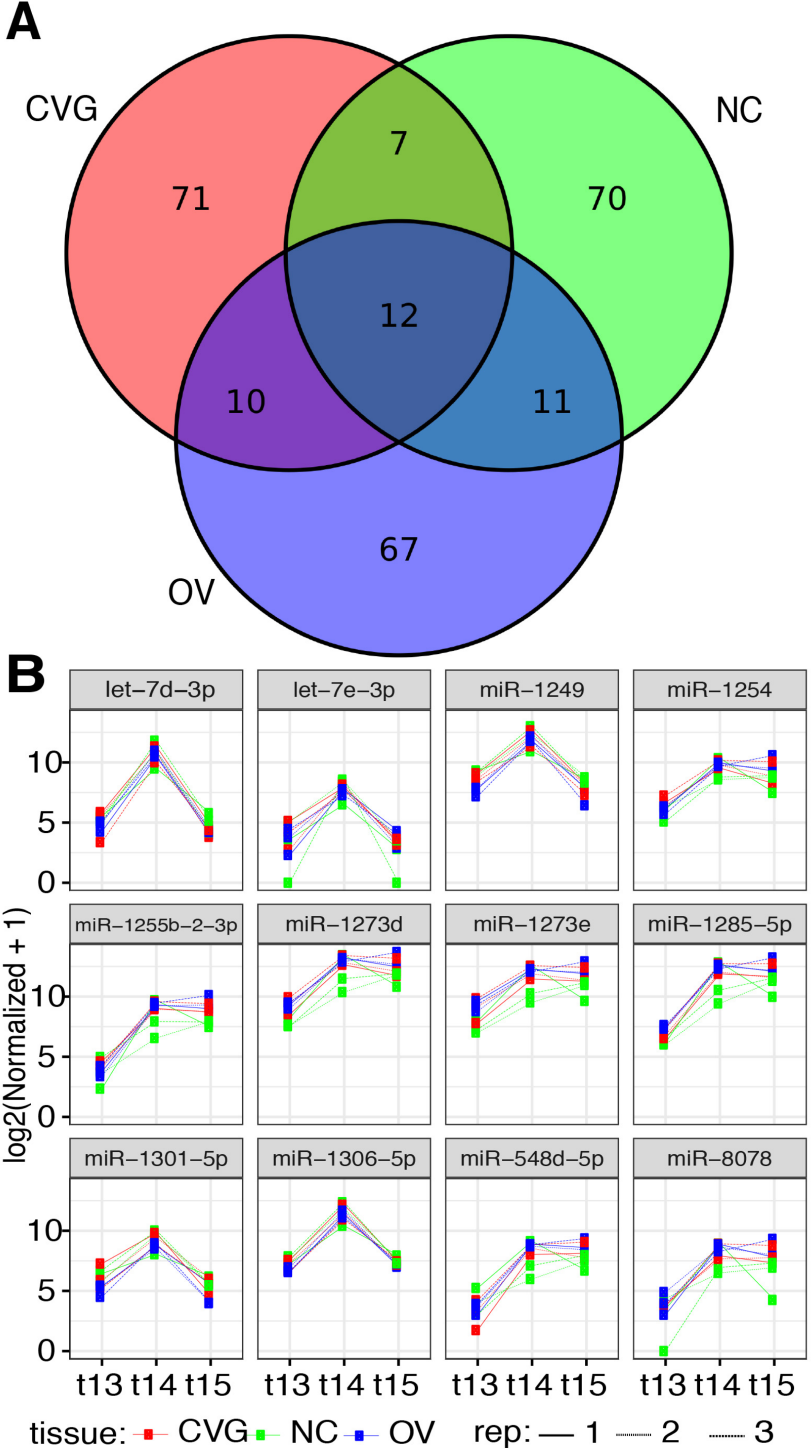
Differentially expressed miRNA across time points. (A) A Venn diagram is presented showing the top 100 miRNAs differentially expressed among the three time points (stages 13, 14, and 15) for each tissue type (CVG, NC, and OV). (B) Normalized counts are plotted for 12 commonly expressed miRNAs that are differentially expressed across time for all three tissues.

The top-100 differentially expressed miRNAs in CVG, OV, and NC, and the overlap across tissues, are summarized in Fig 3A as a Venn diagram. A total of twelve miRNAs were found to be commonly regulated over time in these three tissues; let-7d, let-7e, miR-1249, miR-1254, miR-1255, miR-1273, miR-1285, miR-1301, miR-1306, miR-548, and miR-8078. Differential expression analyses at three developmental time points on these twelves miRNAs are shown in Fig 3B. The complete set of raw data obtained from HTG Edgeseq miRNA WTA, Quality control files, Brain RNA correction, and normalized data are shown in the S1 Dataset. Complete spreadsheets of differential miRNA expression for each tissue comparison and time point are available in the S2 and S3 Datasets, respectively.

**Fig 4:**
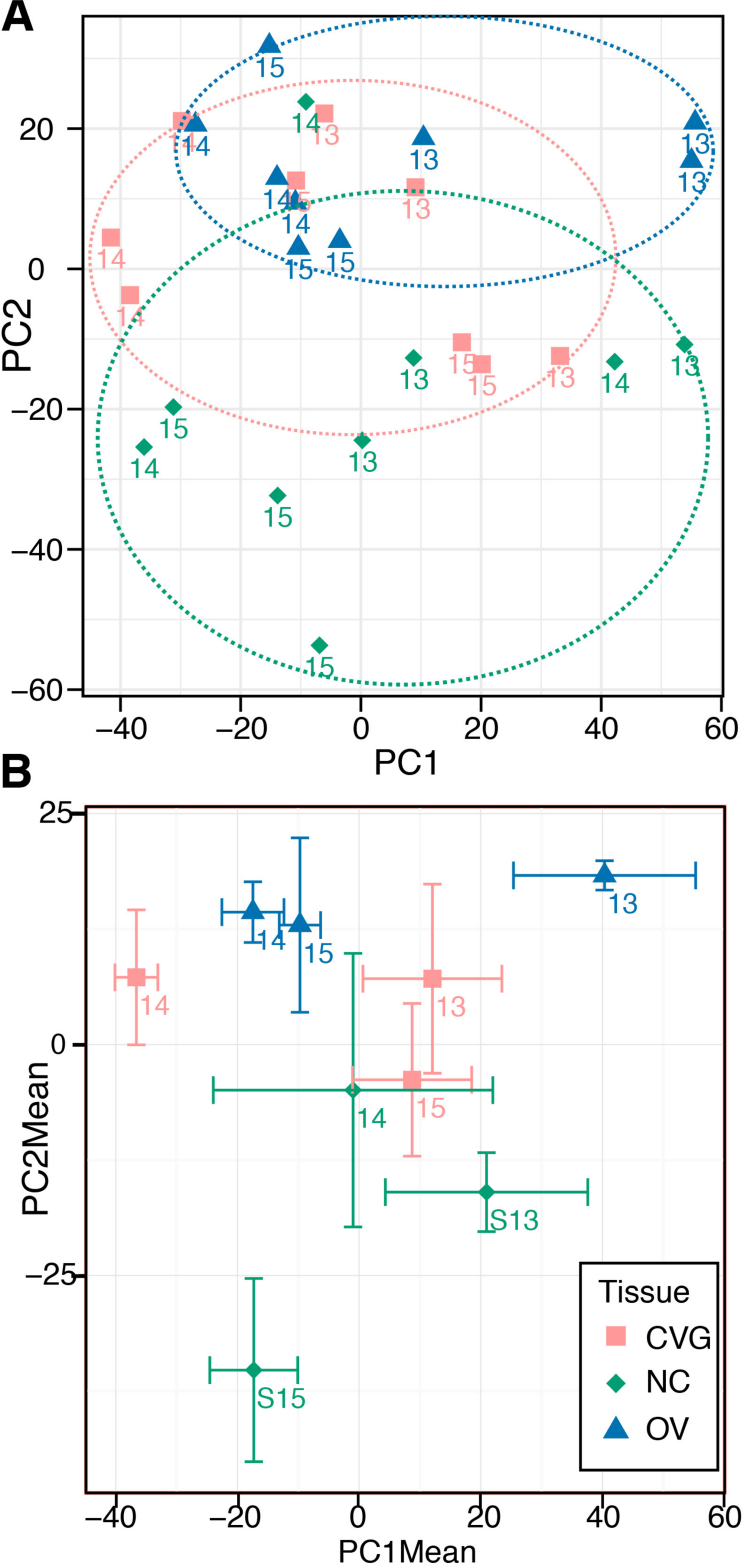
Principal component analysis. (A) Principle components 1 and 2 are plotted for the 27 collected datasets. Loose clustering by tissue type is indicated with dashed colored circles. (B) As an alternative visualization, triplicate measures for each individual tissue/time point set are averaged and plotted with x- and y-standard error bars.

Principal component analysis (PCA) was performed to visualize potential clustering among samples by either tissue or time point. Principal components 1 and 2 are plotted against one another in Fig 4. Although there was no tight clustering among samples by tissue or time point, PC2 is moderately associated with tissue type (Fig 4A, dashed circles). Components are alternatively visualized as the means among technical replicates for a given tissue type and time point with x and y standard error bars (Fig 4B).

### Identification of over-connected transcription factors

**Fig 5:**
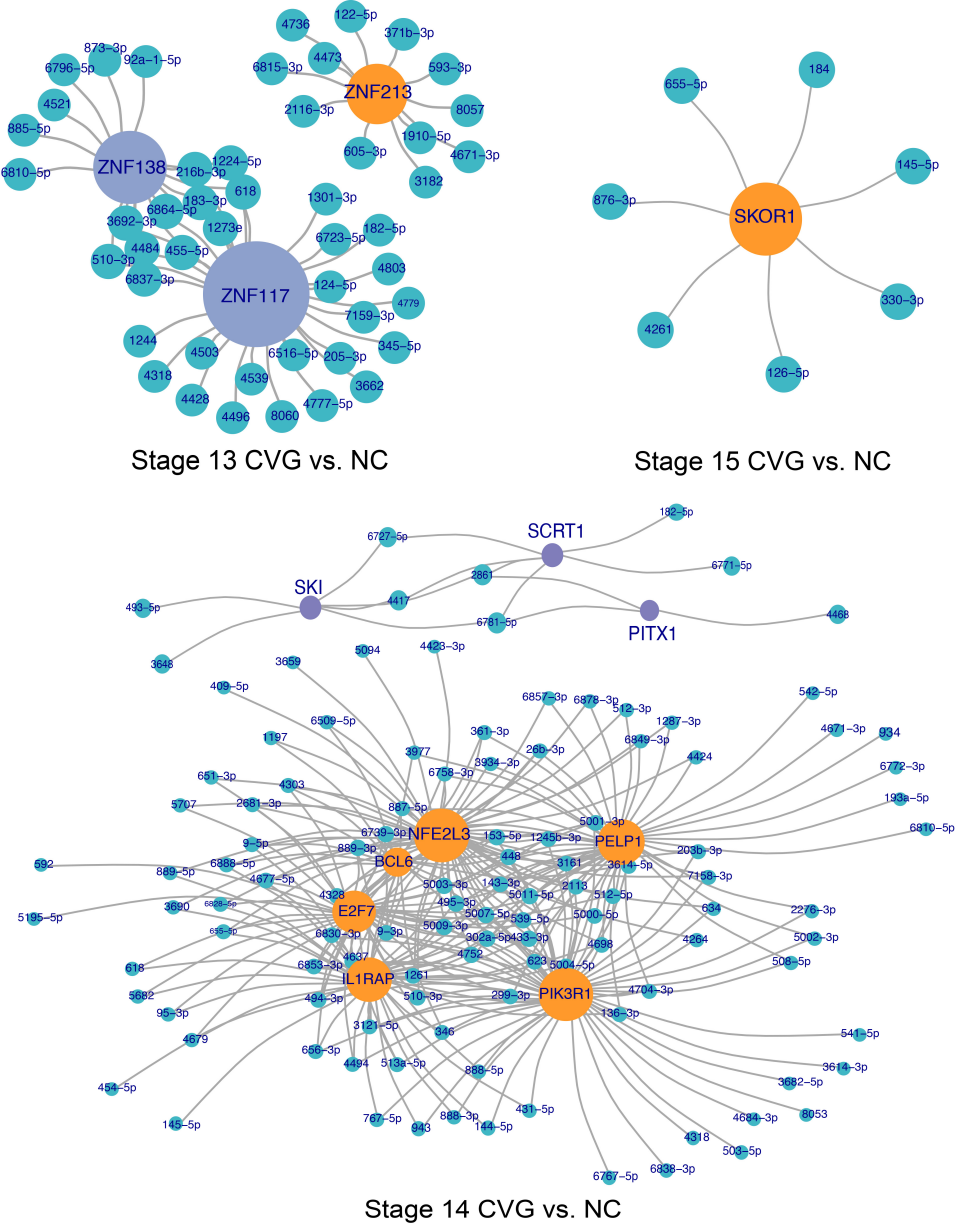
Transcription factor targeting network plots, CVG vs NC comparison. Network plots showing differentially targeted transcription factors between CVG and NC tissues. Green nodes represent differentially expressed miRNAs, while orange and purple nodes represent significantly (false discovery adjusted *p* < 0.10) targeted transcription factors more targeted in either CVG (purple) or NC (orange) tissue. Transcription factor node size is proportional to the number of differentially expressed miRNAs targeting the gene.

**Fig 6:**
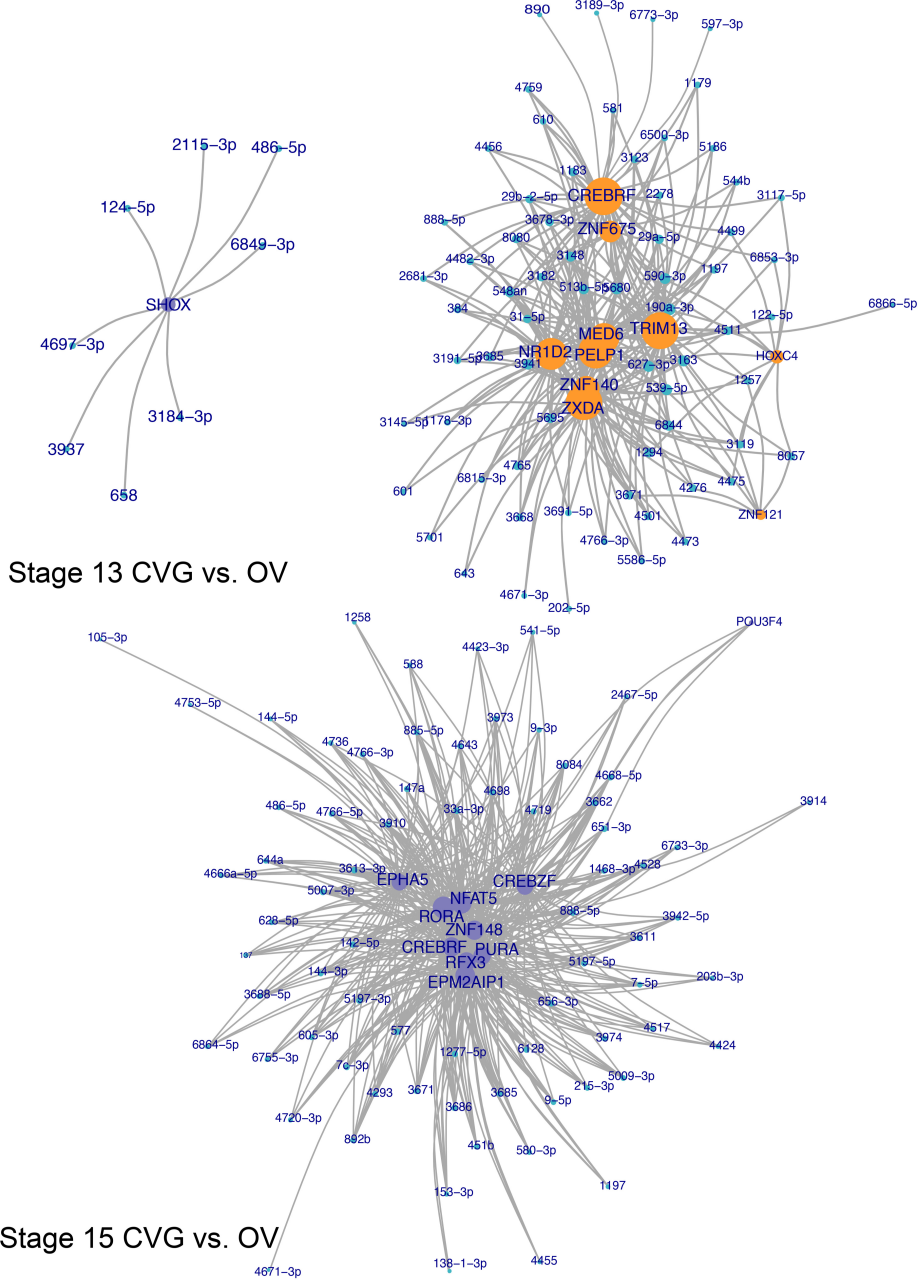
Transcription factor targeting network plots, CVG vs OV comparison. Network plots showing differentially targeted transcription factors between CVG and OV tissues. Green nodes represent differentially expressed miRNAs, while orange and purple nodes represent significantly (false discovery adjusted *p* < 0.10) targeted transcription factors more targeted in either CVG (purple) or OV (orange) tissue. Transcription factor node size is proportional to the number of differentially expressed miRNAs targeting the gene. Only results for stages 13 and 15 are shown, as there are too many significant factors to effectively visualize at stage 14.

To identify differential targeting of transcription factors, genes over-connected to miRNA differential expression datasets were identified by hypergeometric enrichment. The top 10 more-targeted and top 10 less-targeted transcription factors, ranked by significance and filtered for FDR adjusted at *p* < 0.10, are given for CVG vs. NC and CVG vs. OV comparisons for each time point in Tables 1 and 2, respectively. To visualize miRNA targeting of differentially targeted transcription factors, network plots were formed showing transcription factors differentially targeted between CVG and NC tissues (Fig 5) and between CVG and OV tissues (Fig 6), as determined by hypergeometric enrichment. Complete data sets on over-connected transcription factors are included in the S4 Dataset. Note that, in the group column of the Tables 1and 2, “up” indicates more targeting of a gene by miRNA in the case (CVG) vs the comparison tissue (NC or OV), and presumably downregulation of the gene itself. In the CVG vs. NC comparison, zinc finger transcription factors (ZNF213, ZNF117, and 138) at stage 13; PELP1 (EGF), PIK3R1 (FGF), SCRT1 (Zinc finger transcription factor), and SKI (TGF-β signaling) at stage 14; and SKOR1 (SMAD binding (inhibiting BMP signaling)) at stage 15 are likely to relevant for CVG development based on prior literature [43,44]. In the CVG vs. OV comparison, CARF (BDNF transcription), EPHA5 (axonal genesis and synapse guidance), LRP6 (Wnt/beta-catenin signaling) at stage 13; and BBS7 (Sonic hedgehog (SHH) signaling) at stage 14 are similarly relevant for CVG development.

**Table 1:** Differentially targeted transcription factors, CVG vs NC comparison.

Gene Symbol	Group	*p* value	Adjusted *p* value	Notes
ZNF213	Down, Stage 13	1.70E-06	1.64E-02	Zinc finger transcription factor; controls neurogenesis
ZNF117	Up, Stage 13	9.29E-06	2.99E-02	Zinc finger transcription factor; controls neurogenesis
ZNF138	Up, Stage 13	1.10E-07	2.13E-03	Zinc finger transcription factor; controls neurogenesis
BCL6	Down, Stage 14	8.53E-05	3.66E-02	Zinc finger transcription factor; controls neurogenesis through NOTCH-dependent transcriptional complexes at targets
E2F7	Down, Stage 14	3.20E-04	8.58E-02	Directly repressed classical E2F transcription factors such as E2F1
IL1RAP	Down, Stage 14	5.62E-05	2.78E-02	May be involved in IL1B-potentiated NMDA-induced calcium influx in neurons
NFE2L3	Down, Stage 14	9.33E-05	3.83E-02	Leucine zipper transcription factor
PELP1	Down, Stage 14	1.83E-04	6.19E-02	Interacts with growth factor signaling components EGFR
PIK3R1	Down, Stage 14	1.06E-07	2.92E-04	Role in signaling in response to FGFR1, FGFR2, FGFR3, FGFR4
PITX1	Up, Stage 14	1.19E-04	8.49E-02	Identity/structure of hindlimb; DAG and IP3 signaling
SCRT1	Up, Stage 14	1.09E-05	1.97E-02	Zinc finger transcription factor; promotes neural differentiation
SKI	Up, Stage 14	3.35E-05	3.57E-02	Receptor of TGF-β signaling; neural tube development
SKOR1	Down, Stage 15	1.11E-05	3.05E-02	SMAD binding (inhibiting BMP signaling)

down: transcription factors less targeted by miRNA; up: transcription factors more targeted by miRNA; stage: the Carnegie embryonic stage. GeneCards® (www.genecards.org) was used for Notes [45].

**Table 2:** Differentially targeted transcription factors, CVG vs OV comparison.

Gene Symbol	Group	*p value*	Adjusted *p* value	Notes
ZNF121	Down, Stage 13	1.63E-07	2.43E-04	Transcription regulation
TRIM13	Down, Stage 13	2.12E-06	1.41E-03	Involved in turnover of membrane and secretory proteins from the endoplasmic reticulum; signal transducer activity
MED6	Down, Stage 13	7.83E-06	3.63E-03	Transcription cofactor
NR1D2	Down, Stage 13	2.55E-05	8.95E-03	Transcriptional repressor regulating genes involved in circadian rhythm, metabolic function, and inflammatory response
PELP1	Down, Stage 13	5.66E-05	1.40E-02	Interacts with growth factor signaling components EGFR
HOXC4	Down, Stage 13	6.78E-05	1.56E-02	Developmental gene related to anterior-posterior positioning
ZXDA	Down, Stage 13	7.08E-05	1.59E-02	Promotes transcription of MHC class I and II genes
ZNF140	Down, Stage 13	1.00E-04	2.05E-02	Transcriptional repressor
CREBRF	Down, Stage 13	1.20E-04	2.39E-02	Negative regulator of endoplasmic reticulum stress response by CREB3 regulation
ZNF675	Down, Stage 13	1.41E-04	2.67E-02	Transcriptional regulation; involved in osteoclast differentiation by modulating TRAF6 signaling
SHOX	Up, Stage 13	4.89E-05	7.87E-02	Controls growth and development
CREBRF	Up, Stage 15	3.49E-09	8.42E-06	Negative regulator of endoplasmic reticulum stress response by CREB3 regulation
RORA	Up, Stage 15	1.63E-07	1.43E-04	Key regulator of differentiation, development, immunity, circadian rhythm; regulates SHH expression and calcium-mediated signal transduction
PURA	Up, Stage 15	2.91E-07	2.01E-04	Transcription activator
ZNF148	Up, Stage 15	5.50E-07	3.43E-04	Transcriptional regulator; mutations associated with global developmental delay
NFAT5	Up, Stage 15	7.85E-07	4.74E-04	T-cell related transcription factor, immune response
CREBZF	Up, Stage 15	1.14E-06	5.32E-04	Suppresses transcriptional activation by CREB3
EPM2AIP1	Up, Stage 15	2.37E-06	7.84E-04	Binds laforin, function unknown; mutations associated with epilepsy
EPHA5	Up, Stage 15	2.86E-06	8.37E-04	Mediates nervous system development, role in axon guidance and synaptogenesis
POU3F4	Up, Stage 15	2.82E-06	8.37E-04	Neural transcription factor, plays a role in inner ear development
RFX3	Up, Stage 15	3.40E-06	9.66E-04	Transcriptional activator, involved in differentiation during embryogenesis

down: transcription factors less targeted by miRNA; up: transcription factors more targeted by miRNA; stage: the Carnegie embryonic stage. GeneCards® (www.genecard.org) was used for Notes [45].

### Gene set enrichment analysis

To understand tissue specific gene expression profiles that are important to human inner ear development, differentially expressed pathways were determined for each tissue comparison and time point based on miRNA differential expression datasets. The top 16 up- and down-regulated pathways, ranked by significance, are included in Table 3 (CVG vs. NC) and 4 (CVG vs. OV), respectively. Note that a positive log odds ratio implies more miRNA targeting of a pathway, and therefore presumably inhibition of the pathway itself. In the CVG vs. NC comparison, the Dicer pathway, Insulin growth factor-1 (IGF-1) pathway, activated point mutants of FGFR2, and regulation of commissural axon pathfinding by SLIT and ROBO are likely relevant for CVG development (Table 3), because they have negative log odds ratios, and are thus less likely to be inhibited by miRNAs and more likely to be enriched in the ganglion as compared to the NC. In the CVG vs. OV comparison, regulation of commissural axon pathfinding by SLIT and ROBO is once again likely relevant for CVG development (Table 4). Complete differential pathway analyses data are included in the S5 Dataset.

**Table 3:** Enriched gene sets, CVG vs NC comparison.

Pathway	LOR	*p* value	Adjusted *p* value	Time point
Dicer pathway	-0.32	4.06E-09	1.35E-04	Stage 13
Mitotic anaphase	-0.31	2.56E-06	3.43E-02	Stage 13
Trafficking of myristoylated proteins to the cilium	-0.26	3.09E-06	3.43E-02	Stage 13
Inhibition of cellular proliferation by GLEEVEC	-0.26	7.61E-06	6.34E-02	Stage 13
Metastatic brain tumor	-0.43	2.34E-05	1.56E-01	Stage 13
Zinc efflux and compartmentalization by the SLC30 family	-0.21	8.61E-05	4.65E-01	Stage 13
A tetrasaccharide linker sequence is required for GAG synthesis	-0.22	9.81E-05	4.65E-01	Stage 13
Teniposide Action Pathway	-0.26	1.32E-04	4.65E-01	Stage 13
Teniposide Metabolism Pathway	-0.26	1.32E-04	4.65E-01	Stage 13
Butanoate metabolism	-0.26	1.40E-04	4.65E-01	Stage 13
IGF-1 signaling pathway	-0.20	1.76E-04	5.11E-01	Stage 13
Prostaglandin formation from arachidonate	-0.26	1.97E-04	5.11E-01	Stage 13
RIG-I-like receptor signaling pathway	-0.24	2.07E-04	5.11E-01	Stage 13
Constitutive signaling by AKT1 E17K in cancer	-0.21	2.15E-04	5.11E-01	Stage 13
Hyaluronan biosynthesis and export	-0.27	2.61E-04	5.81E-01	Stage 13
*S*-methyl-5-thio-&alpha;-D-ribose 1-phosphate degradation	-0.25	3.53E-04	6.86E-01	Stage 13
Plasmalogen biosynthesis	-0.27	1.25E-06	4.17E-02	Stage 14
Activated point mutants of FGFR2	-0.29	3.90E-06	6.49E-02	Stage 14
Spermine biosynthesis	-0.21	6.94E-05	5.27E-01	Stage 14
Formaldehyde oxidation	-0.21	7.82E-05	5.27E-01	Stage 14
Heart development	-0.21	1.76E-04	5.27E-01	Stage 14
Synthesis of CL	-0.21	1.96E-04	5.27E-01	Stage 14
Copper homeostasis	-0.22	1.98E-04	5.27E-01	Stage 14
NR1D1 (REV-ERBA) represses gene expression	-0.21	2.02E-04	5.27E-01	Stage 14
Pyrimidine deoxyribonucleotides *de novo* biosynthesis	-0.25	2.03E-04	5.27E-01	Stage 14
Ion transport by P-type ATPases	-0.23	2.16E-04	5.27E-01	Stage 14
Adenine and adenosine salvage II	-0.24	2.24E-04	5.27E-01	Stage 14
Interleukin-6 signaling	-0.20	2.30E-04	5.27E-01	Stage 14
Synthesis of UDP-N-acetyl-glucosamine	-0.20	2.34E-04	5.27E-01	Stage 14
UDP-*N*-acetyl-D-glucosamine biosynthesis II	-0.20	2.34E-04	5.27E-01	Stage 14
Regulatory RNA pathways	-0.25	2.38E-04	5.27E-01	Stage 14
Regulation of commissural axon pathfinding by SLIT and ROBO	-0.21	2.55E-04	5.30E-01	Stage 14
Fatty acid elongation—saturated	-0.26	1.28E-05	4.26E-01	Stage 15
Hypusine biosynthesis	-0.44	6.54E-04	1.00E+00	Stage 15
Doxorubicin pathway (cancer cell), pharmacodynamics	0.23	7.66E-04	1.00E+00	Stage 15

LOR: Logarithm Odds Ratio.

**Table 4:** Enriched gene sets, CVG vs OV comparison.

Pathway	LOR	*p* value	Adjusted *p* value	Time point
Fatty acid elongation—saturated	0.25	2.44E-05	5.92E-01	Stage 13
Synthesis and processing of ENV and VPU	0.29	3.55E-05	5.92E-01	Stage 13
Synthesis of UDP-*N*-acetyl-glucosamine	-0.20	1.33E-04	9.94E-01	Stage 13
UDP-*N*-acetyl-D-glucosamine biosynthesis II	-0.20	1.33E-04	9.94E-01	Stage 13
Acyl chain remodeling of DAG and TAG	0.21	1.49E-04	9.94E-01	Stage 13
ERK1/ERK2 MAPK signaling pathway	0.22	1.86E-04	1.00E+00	Stage 13
GDP-mannose biosynthesis	0.22	3.99E-04	1.00E+00	Stage 13
FGFR4 mutant receptor activation	0.35	4.28E-04	1.00E+00	Stage 13
Degradation of the RAR and RXR by the proteasome	0.20	4.35E-04	1.00E+00	Stage 13
Propionyl-CoA catabolism	-0.23	6.89E-04	1.00E+00	Stage 13
Microtubule-dependent trafficking of connexons from Golgi to the plasma membrane	-0.23	9.93E-04	1.00E+00	Stage 13
Fatty acid elongation—saturated	0.26	8.88E-06	1.48E-01	Stage 14
Reactions specific to the hybrid N-glycan synthesis pathway	0.24	1.38E-05	1.54E-01	Stage 14
Acyl chain remodeling of DAG and TAG	0.22	5.59E-05	4.65E-01	Stage 14
Saturated fatty acids beta-oxidation	0.31	7.24E-05	4.83E-01	Stage 14
Synthesis and processing of ENV and VPU	0.27	8.97E-05	4.98E-01	Stage 14
Vitamins B6 activation to pyridoxal phosphate	0.19	2.67E-04	1.00E+00	Stage 14
ChREBP activates metabolic gene expression	0.23	4.16E-04	1.00E+00	Stage 14
Synthesis of GDP-mannose	0.21	5.88E-04	1.00E+00	Stage 14
Degradation of the RAR and RXR by the proteasome	0.19	7.54E-04	1.00E+00	Stage 14
Arachidonate production from DAG	0.19	9.31E-04	1.00E+00	Stage 14
Activation of CSK by camp-dependent protein kinase inhibits signaling through the T-cell receptor	0.21	9.70E-04	1.00E+00	Stage 14
*S*-adenosyl-*L*-methionine biosynthesis	0.18	9.72E-04	1.00E+00	Stage 14
Macroautophagy	0.33	1.37E-07	4.56E-03	Stage 15
Plasmalogen synthesis	0.32	1.33E-06	1.57E-02	Stage 15
BMI1	0.36	2.40E-06	1.57E-02	Stage 15
Excitatory neural signaling through 5-HTR 7 and serotonin	0.26	2.74E-06	1.57E-02	Stage 15
The 41bb-dependent immune response	0.25	3.02E-06	1.57E-02	Stage 15
Mitochondrial transcription initiation	0.27	3.29E-06	1.57E-02	Stage 15
Regulation of commissural axon pathfinding by SLIT and ROBO	0.27	3.29E-06	1.57E-02	Stage 15
Phosphorylation of proteins involved in the G2/M transition by Cyclin A: CDC2 complexes	0.37	4.05E-06	1.69E-02	Stage 15
D4GDI signaling pathway	0.32	5.05E-06	1.87E-02	Stage 15
Herpes simplex infection	0.30	6.33E-06	2.11E-02	Stage 15
Early phase of HIV life cycle	0.27	9.16E-06	2.77E-02	Stage 15
Plasmalogen biosynthesis	0.24	1.06E-05	2.88E-02	Stage 15
DAP12 interactions	0.25	1.13E-05	2.88E-02	Stage 15
Hypoxia-mediated EMT and stemness	0.32	1.25E-05	2.95E-02	Stage 15
Gefitinib Pathway, Pharmacokinetics	0.27	1.33E-05	2.95E-02	Stage 15

LOR: Logarithm Odds Ratio.

**Fig 7:**
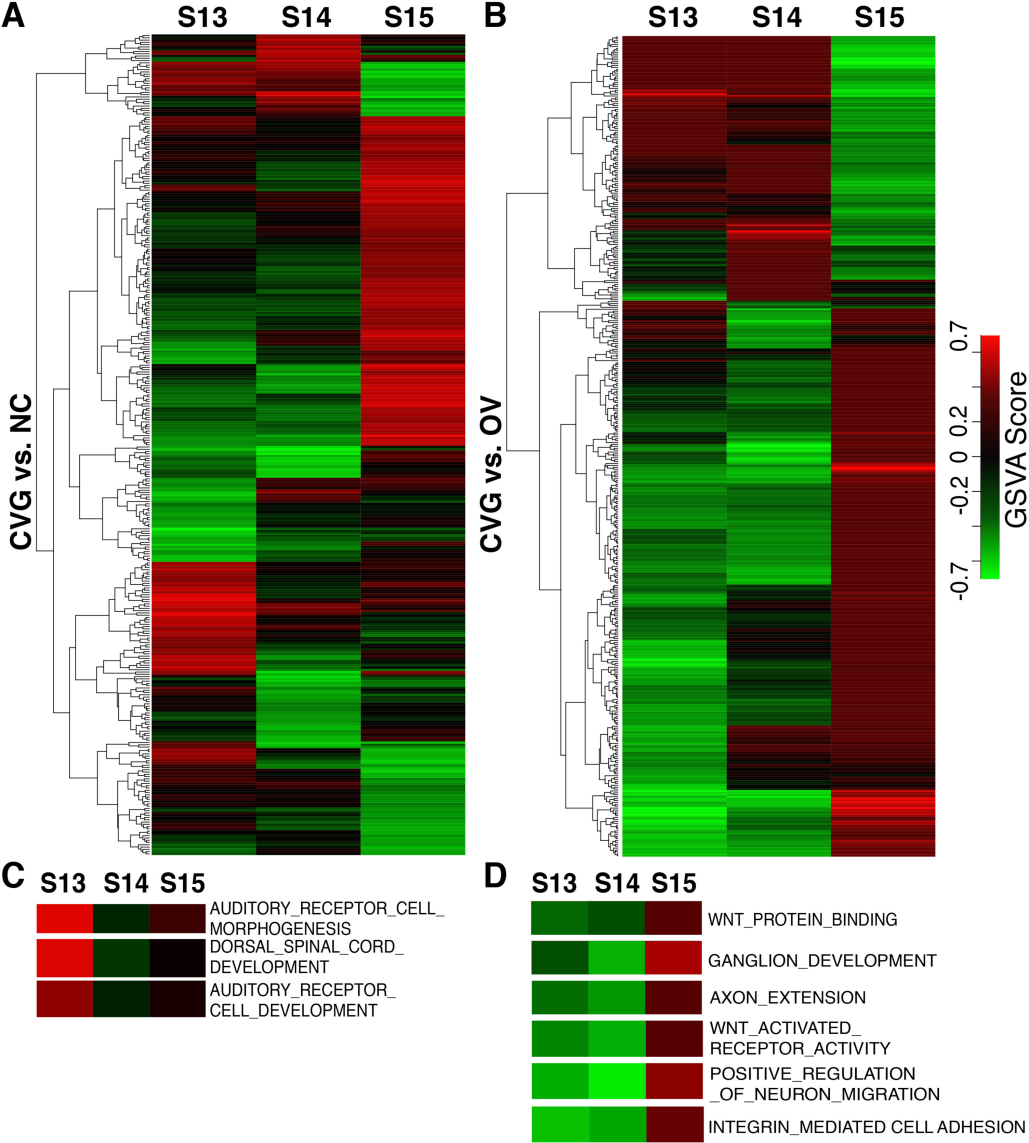
Differentially activated gene ontological pathways by GSVA analysis. Heatmap comparison of CVG with NC (A), and of CVG with OV (B). Each column represents the sample indicated at the top (three time points), each row represents an identified GO Term pathway (see the complete set in the S6 Dataset). The expression levels of GSVA scores are depicted according to the color scale (middle right). Red or green indicate expression levels above or below the median, respectively. The magnitude of deviation from the median is represented by color saturation. Heatmaps representing the GSVA score of GO term pathways relevant to CVG development that are significantly modulated in CVG. vs. NC comparison (C) and CVG vs. OV (D).

**Fig 8:**
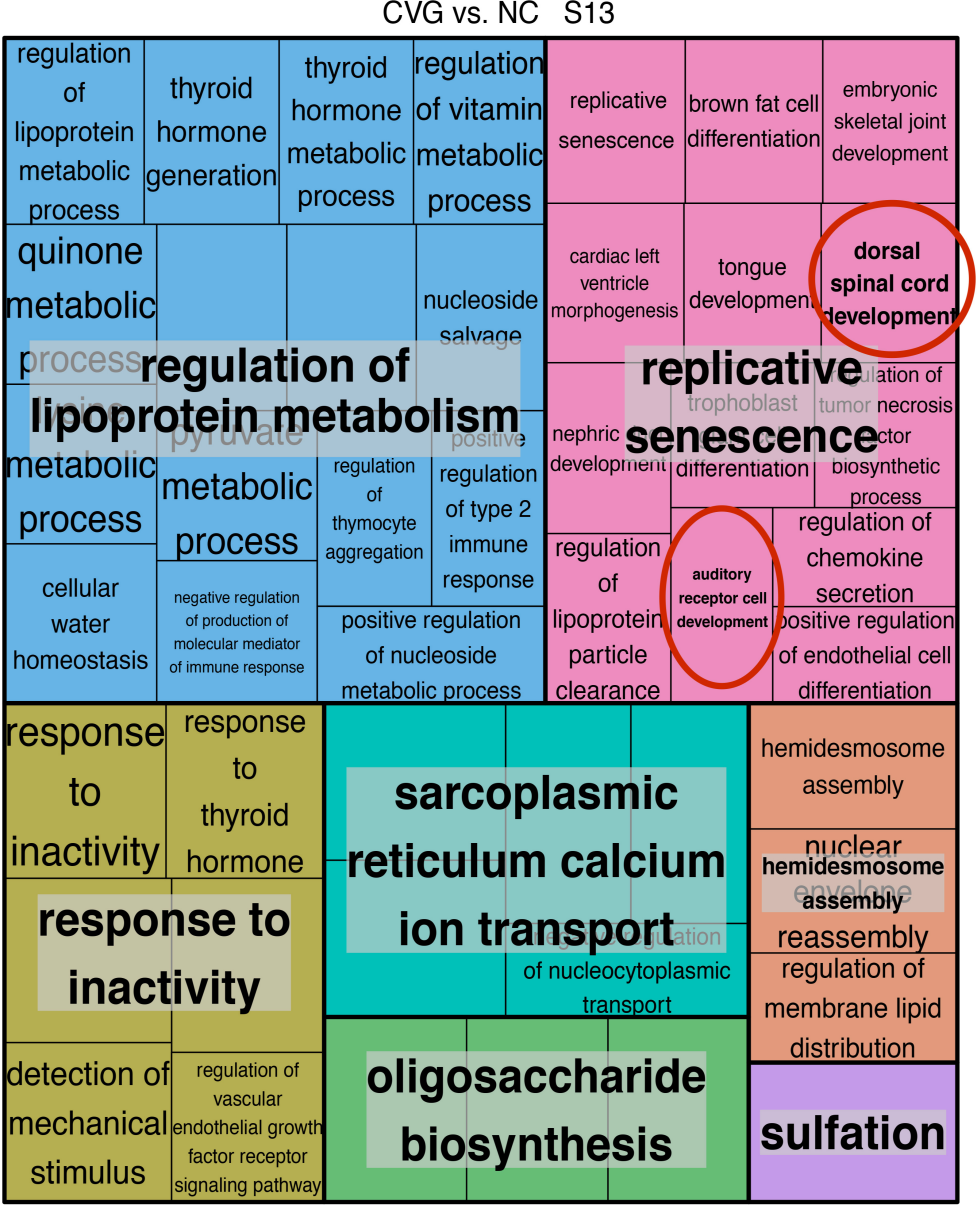
REVIGO treemap of relevance similarity analysis on enriched pathways comparing CVG vs. NC (stage 13). REVIGO treemap summarizing Gene Ontology biological process categories over-represented in CVG cells compared to NC cells at stage 13. All terms are included with a FDR adjusted *p*-value cutoff at 0.05 from the enrichment analysis. The relevance similarity C-score (uniqueness) cut-off is chosen at 0.7. The size of each rectangle is proportional to the uniqueness for that category. Red circles indicate relevant CVG-development pathways.

Since each method for performing pathway analysis can have method dependent bias, we additionally performed a non-parametric analysis using the concept of gene set enrichment analysis [33] on the gene expression profile predicted from the miRNA expression pattern in CVG, OV and NC. From this analysis, we again identified functional gene sets defined in Gene Ontology terms that are positively or negatively enriched in each tissue type at different time points (Fig 7). Fig 7 shows two signatures of the differentially activated Gene Ontology pathways by GSVA analysis in CVG vs. NC comparison (Fig 7A) and CVG vs. OV (Fig 7B) in heatmap format. Using this method, we found that different numbers of pathways were identified as significantly enriched when comparing different tissue types at different stages of development (S5 Fig), with stage 15 having highest number of enriched pathways (209 pathways) when comparing CVG to NC or OV. To generate a signature, any pathway that was significantly enriched either positively or negatively at *p*-<0.05 in at least one time point was included in the heatmap. Note that there are many GO term pathways directly relevant to CVG development (Fig 7C and 7D). These include Wnt activated receptor activity, ganglion development, axon extension, positive regulation of neuron migration, and integrin mediated cell adhesion (higher GSVA score in Stage 15) at different time points (Fig 7D) while most common category of Gene Ontology terms for all comparisons are pathways involved in general biological processes (shown in blue bars in S5 Fig). To better focus on the biological processes that are enriched in different tissue types, we additionally performed the REVIGO analysis described in Supek et al., removing pathways that are functionally redundant after analyzing Gene Ontology classification [46] (Fig 8). Specific themes and processes were identified comparing Gene Ontology terms identified in CVG when comparing to NC at stage 13 (Fig 8) and at stage 14 (S6 Fig). For example, six overarching biological processes were identified by examining enriched pathways at developmental stage 13 when comparing CVG to NC: regulation of lipoprotein metabolism, oligosaccharide biosynthesis, response to inactivity, hemidesome assembly, sarcoplasmic reticulum calcium ion transport, and replicative senescence. Note that replicative senescence includes pathways that are likely to relevant to CVG development such as auditory cell development and dorsal spinal cord development (red circled in Fig 8). A complete list of all enriched pathways is available in the S6 Dataset.

### Computational prediction of the miR-183 family targeted genes

To further investigate how the miR-183 family targeted genes are expressed in the inner ear (CVG) development in humans, we computationally predicted miR-183 family targeted genes using TargetScanHuman (release 7.1). More than 1,000 genes were identified (complete datasets are included in S7 Dataset). The Deafness Variation Database (version 8.0) was then used to identify the targets that are associated with human hereditary hearing loss and deafness. Table 5 shows the list of predicted miR-183 family target genes that are relevant to human hereditary hearing loss and deafness.

**Table 5:** Predicted target genes for the MiR-183 family using TagetScanHuman and a Deafness Variation Database (version 8.0).

miRNA	Predicted target genes
miR-183 family	*MITF*, *TECTB*, *EDNRB*, *CD164*, *OSBPL2*, *MET*, *CLIC5*, *GRHL2*, *COL4A4*, *TBX1*, *RDX*, *S1PR2*, *CCDC50*, *PNPT1*, *PEX6*, *SLC26A4*, *OTOG*, *COL9A1*, *COL4A6*, *PTPRQ*

## Discussion/conclusions

Due to inaccessibility of human inner ear tissue, prior miRNA expression studies have been limited to animals, and in particular, mouse models [10,47–50]. Further, the standard use in the United States and elsewhere of suction curettage (51), which generally renders embryonic structures unidentifiable, now makes it difficult even to obtain human tissue for study. However, the development of techniques for analyzing miRNA expression, along with the FFPE specimens provided by the Bruska group, enabled us to perform these novel analyses of human embryonic tissues. Note that due to the small sample size, our results should be cautiously assessed; bearing in mind that differential expression across time points can be confounded by biological noise, while differences among tissues could be specific to the sampled individual.

The differential expression of miR-183 family members (miR-96-5p, miR-182-5p, and miR-183-5p) in human CVG cell types at stage 13 correlates well with prior studies in mice. Weston et al. found that expression of the triad of miR-96, miR182, and miR-183 during development is relatively restricted to mouse inner ear compared to brain, heart, and whole embryo [3]. Sacheli et al. noted the earliest expression of miR-183 and miR-182 in the mouse otic vesicle at embryonic day 9. (E9.5), expression of all three miRNAs in OV, CVG, and neural tube at E11.5, and limited expression was observed at E14.5 [51]. By P0, this triad was strongly expressed in hair cells of the cochlea and vestibular system, as well as in the SGNs. We found that in human CVG, the miRNA-183 family was differentially expressed at stage 13 (E11), but only miR-182-5p was expressed at stage 14 (E11.5), and the miRNA family was not differentially expressed at stage 15 (E12). This may reflect differences between mouse and human development, but the small sample size in this study limits firm conclusions. Regardless, our findings suggest that the miR-183 family is important in human as well as murine inner ear development.

Twelve miRNAs out of the 100 most differentially expressed miRNAs across three time points were commonly expressed in all three tissues as presented in our Venn diagram (Fig 3). The majority of the common miRNAs (let-7 family, miRNA 548, 1255, 1273, 1285, 1301, and 1306) are involved in developmental timing, cell proliferation, cell-cycle regulation, and apoptosis [52,53]. Also, miR-1254 is known to regulate the epithelial to mesenchymal transition (EMT) [54]. EMT plays an important role in the delamination and escape through into the mesenchyme of both neural crest cells and placodal sensory neurons such as CVG [55]. The commonality of expression of these twelve miRNAs among the CVG, OV, and NC cell types suggests that these miRNAs are involved in both inner ear and neural crest development.

The over-connected transcription factor analysis revealed a number of transcription factors that are known to be involved in CVG development in mice. For example, SKI was found to be upregulated in the CVG. vs. NC comparison, indicating more inhibition of SKI by miRNA in the CVG. SKI family transcription factors have been known to downregulate TGF-β signaling in mice thereby promoting neuronal induction including in the CVG [56]. CARF (BDNF transcription, stage 13), EPHA5 (axonal genesis and synapse guidance, stage 13), LRP6 (Wnt/beta-catenin signaling, stage 13), and BBS7 (Sonic hedgehog (SHH) signaling, stage 14) have been previously shown to be relevant in mouse CVG development [57]. Other transcription factors identified by our analysis could represent important developmental effectors that have not been identified previously; our data thus has the potential to generate a series of follow up experiments to investigate development in the CVG, NC, and OV.

Our initial gene set enrichment analysis revealed that the Dicer, IGF-1, and FGFR2 pathways (stage 13) and regulation of commissural axon pathfinding by SLIT and ROBO (stage 14 and 15) were relevant ontogenetic pathways for CVG development. The Dicer pathway was the most statistically significant ontogenetic pathway at stage 13 in humans. A previous study in the mouse inner ear has shown that progressive reduction of miR-183 expression after *Pax2*-Cre conditional *Dicer* knockout (KO) results in progressive loss of neurosensory gene expression, arrested neurosensory development and loss of CVGs with an associated disruption of morphogenesis [4]. More recently, a *Dicer1* conditional KO line (*Atoh1-cre;Dicer1* ^*flox/flox*^ *and Foxg1-cre; Dicer1* ^*flox/flox*^) showed defects in proliferation in the prosensory domain of the cochlea including CVGs [7,8,58]. Our observations coupled with the findings in mice suggest that Dicer pathway interactions with the miR-183 family play an essential role in the regulation of early mice inner ear development. Similarly, previous studies in mouse demonstrated that FGF2 signaling through FGFR2 and endogenous IGF-1 signaling are necessary for development of SGNs [59–61], consistent with our findings in the embryonic human inner ear.

In addition to identifying novel regulatory pathways derived from differential miRNA expression in specific embryonic inner ear tissues, we found that many of the enriched pathways occur at different stages of inner ear development. While comparing CVG to NC and OV, we observed that many more pathways were significantly enriched at stage 15, but that regulatory pathways of inner ear development, neurogenesis or axis patterning seemed to occur earlier at stages 13 and 14. Specifically, pathways involved in auditory receptor cell morphogenesis as well as many cell fate determination and pattering pathways were enriched in CVG at stages 13 and 14. By contrast, many of the pathways enriched in stage 15 are related to biosynthesis, metabolism and proliferation, suggesting that this is likely an embryonic stage allowing for growth after cell fate determination has already occurred.

Identification of miRNA-targeted genes is one of the most significant elements for understanding the function of a miRNA in the human inner ear development [47]. Among our predicted miR-183 family targeted genes that are related to human inner ear (CVG) development (Table 5), it is worthwhile to mention *TBX1* (T-box domain 1), as a previous study has confirmed *tbx-1* as one of target genes for the conserved miR-183 family in a mouse model [62]. *Tbx1* was required for morphogenesis and growth of the murine otocyst [63]. In humans, *TBX1* is a critical gene in DiGeorge syndrome, with patients presenting phenotypes including inner ear abnormalities, sensorineural hearing loss [64–66], and vestibular loss [67,68]. Therefore, we speculate that the transcriptional regulation of *TBX1* in human may be influenced by the miR-183 family, providing a crucial function in developmental signaling pathways in the human inner ear.

It should be noted that this study is limited by small sample size; for each time point, only one biological specimen was obtained. It is, therefore, plausible that expression differences between time points reflected biological variations (i.e., noise) between individuals rather than tissue-specific variation over time. Differences between tissue types for a given time point could similarly be influenced by individual-specific variation. The small sample size also limits the statistical power to detect differential miRNA expression. While our findings are suitable for hypothesis generation, they should be critically considered on a case-by-case basis, bearing these limitations in mind. Additionally, though we would have liked to validate our findings using secondary methods such as qPCR, these experiments could not be performed due to sample constraints; all available tissue was used to obtain miRNA expression data with adequate quality control from HTG-seq. Many samples yielded only 500–1000 cells upon laser dissection (S1 Table). Despite these limitations, many of our findings correlate well with prior findings in the mouse which suggests that they are truly informative about inner ear development in humans. In addition to providing insight into the mechanisms governing the development of the human inner ear, our findings also identify potential signaling pathways for promoting regeneration of human spiral ganglions and other components of the inner ear using pluripotent stem cells in the future.

## Supporting information

S1 Table Detailed description of the three FFPE samples. C-Stage: the Carnegie developmental stage; CR length: Crown-rump length; NC: neural crest; CVG: cochlear-vestibular ganglions; OV: otic vesicle.
(DOCX)

S1 Fig The quality control chart. SD: standard deviation. The difference between averaged ANT (control) median normalized counts and the grand mean of the averaged ANT values (Δmean) was computed for each sample and plotted to demonstrate statistical process control for HTG measurement techniques. Samples were deemed acceptable if they fell within ±2SD.
(PDF)

S2 Fig Boxplots for expression of the top 20 differentially expressed miRNAs over the three time points for CVG, NC, and OV. Plots of normalized counts versus time for the top 20 differentially expressed miRNAs for each tissue ranked by significance. Error bar = ±2SD, S#1: sample 1; S#2: sample 2; S#3: sample 3.
(PDF)

S3 Fig Heatmap of differentially activated gene ontological pathways by GSVA analysis (CVG vs. NC). (TIFF)

S4 Fig Heatmap of differentially activated gene ontological pathways by GSVA analysis (CVG vs. OV). (TIFF)

S5 Fig Enriched pathways by GSVA analysis. Comparisons among three tissue types at three time points. Blue bar: biological function; orange bar: molecular function; gray bar: cellular component; and yellow bar: others. The number of pathway is shown inside or next to each bar. S: Stage.
(PDF)

S6 Fig REVIGO treemap of relevance similarity analysis on enriched pathways comparing CVG vs. NC (stage 14). REVIGO treemap summarizing Gene Ontology biological process categories over-represented in CVG cells compared to NC cells at stage 14. All terms are included with a FDR adjusted *p*-value cutoff at 0.05 from the enrichment analysis. The relevance similarity C-score (uniqueness) cut-off is chosen at 0.7. The size of each rectangle is proportional to the uniqueness for that category. Red circles indicate relevant neuronal development pathways.
(PDF)

S1 Dataset Raw expression count data. Raw expression data for each sample as provided to us by HTG-seq. Raw counts for each sample are converted to counts per million (CPM), then median normalized in the “Normalized” tab. Spiked in brain RNA is used as a control, expecting high correlation between groups. Samples are matched to their normalized data in the “Groupings” tab, then averaged prior to computing fold changes between tissues in the “Fold Change” tab.
(XLSX)

S2 Dataset Differential expression data across three tissue types. Differential expression data based analysis with the R Bioconductor limma package. The spreadsheet includes averaged normalized miRNA expression values for each tissue, fold changes for all tissue comparisons, p-values, and false discovery adjusted p-values from limma. Each time point is included as a separate tab.
(XLSX)

S3 Dataset Differential expression data across three time points. Differential expression data analyzed using the R Bioconductor timecourse package. The spreadsheet includes averaged normalized miRNA expression values for each time point and the maximum Hotelling T values from the three possible time point comparisons. Results are ordered by rank. Each time point is included as a separate tab.
(XLSX)

S4 Dataset Differentially targeted genes. Complete differential gene targeting analysis for each tissue comparison and time point (separate tabs), as analyzed by hypergeometric enrichment. Complete results are included, as well as filtered results for each comparison at the false discovery adjusted *p* < 0.10 level. Genes that contain “transcription factor” in their Gene Ontology are highlighted in light green.
(XLSX)

S5 Dataset Differentially regulated pathways. Complete gene set analysis results for each tissue comparison and time point (separate tabs), analyzed using the R Bioconductor mdgsa package. All results are included in each tab.
(XLSX)

S6 Dataset Complete set of differentially activated gene ontologically activated pathway analysis by GSVA analysis for each tissue comparison. All results are included in each tab.
(XLSX)

S7 Dataset Complete set of predicted miR-183 family targeted genes using TargetScanHuman (release 7.1). (XLSX)

S1 File Materials and methods. (DOCX)
