# Supplementary material for: Developmental profiling of microRNAs in the human embryonic inner ear
Source: PLoS One. 2018 Jan 26;13(1):e0191452. doi: 10.1371/journal.pone.0191452 (PMC5786302; doi:10.1371/journal.pone.0191452)
Supplement: S1 Table — C-Stage: the Carnegie developmental stage; CR length: Crown-rump length; NC: neural crest; CVG: cochlear-vestibular ganglions; OV: otic vesicle. (DOCX) [file pone.0191452.s001.docx]

| **Catalog Number** | **C-Stage** | **CR Length (mm)** | **Post-gestational Age (days)** | **Plane of Section** | **Number of cells** |
| --- | --- | --- | --- | --- | --- |
| B218 | 13 | 5.5 | 32 | Coronal | NC1 (> 1000 cells)  NC2 (> 1000 cells)  NC3 (> 1000 cells)  CVG1 (> 1000 cells)  CVG2 (> 1000 cells)  CVG3 (> 1000 cells)  OV1 (> 1000 cells)  OV2 (> 1000 cells)  OV3 (> 1000 cells) |
| AS21 | 14 | 7.5 | 33 | Coronal | NC1 (400-500 cells)  NC2 (500-600 cells)  NC3 (400-500 cells)  CVG1 (700-800 cells)  CVG2 (1400-1500 cells)  CVG3 (1500-1600 cells)  OV1 (> 2000 cells)  OV2 (> 2000 cells)  OV3 (> 2000 cells) |
| PJK20 | 15 | 9 | 35 | Coronal | NC1 (400 to 600 cells)  NC2 (300 to 500 cells)  NC3 (300 to 400 cells)  CVG1 (500 to 600 cells)  CVG2 (500 to 1400 cells)  CVG3 (800 to 1100 cells)  OV1 (800 to 900 cells)  OV2 (600 to 1100 cells)  OV3 (900 to 1200 cells) |

**S1 Table. Detailed Description of the three FFPE samples.**

C-Stage: the Carnegie developmental stage; CR length: craniorostal length; NC: neural crest; CVG: cochleovestibular ganglions; OV: otic vesicle
