# Supplementary figures and images for: Developmental profiling of microRNAs in the human embryonic inner ear
Source: PLoS One. 2018 Jan 26;13(1):e0191452. doi: 10.1371/journal.pone.0191452 (PMC5786302; doi:10.1371/journal.pone.0191452)

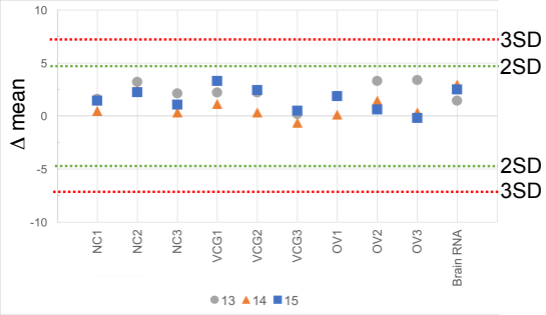

Supplement: S1 Fig — SD: standard deviation. The difference between averaged ANT (control) median normalized counts and the grand mean of the averaged ANT values (Δmean) was computed for each sample and plotted to demonstrate statistical process control for HTG measurement techniques. Samples were deemed acceptable if they fell within ±2SD. (PDF) [file pone.0191452.s002.pdf]

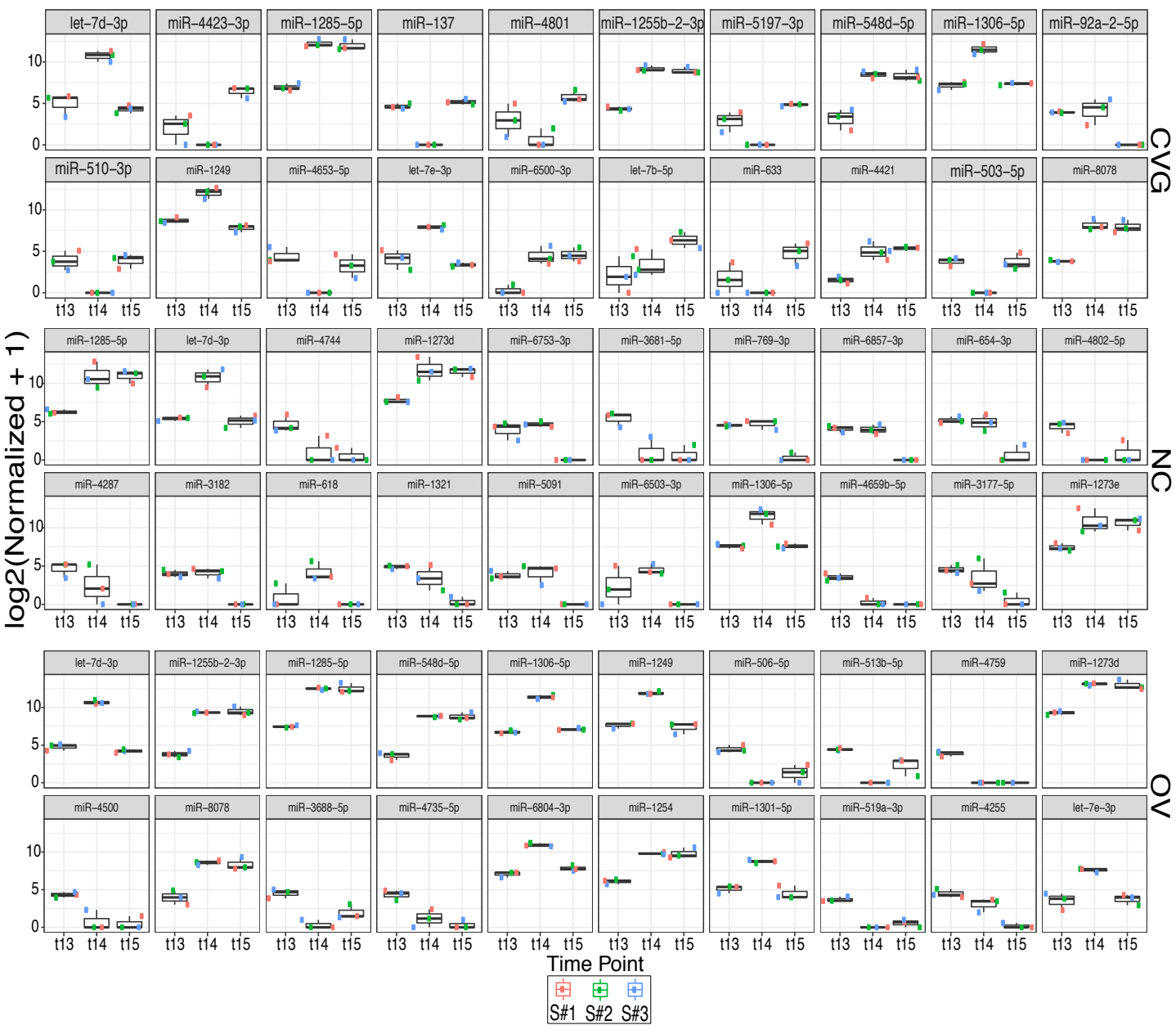

Supplement: S2 Fig — Plots of normalized counts versus time for the top 20 differentially expressed miRNAs for each tissue ranked by significance. Error bar = ±2SD, S#1: sample 1; S#2: sample 2; S#3: sample 3. (PDF) [file pone.0191452.s003.pdf]

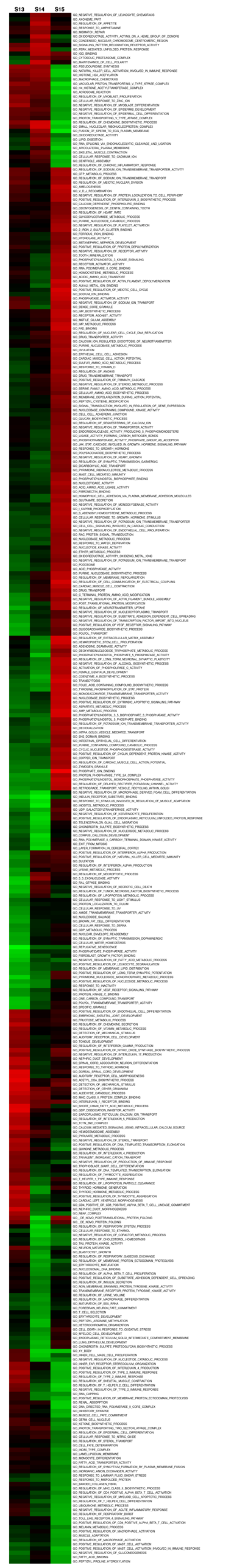

Supplement: S3 Fig — (TIFF) [file pone.0191452.s004.tiff]

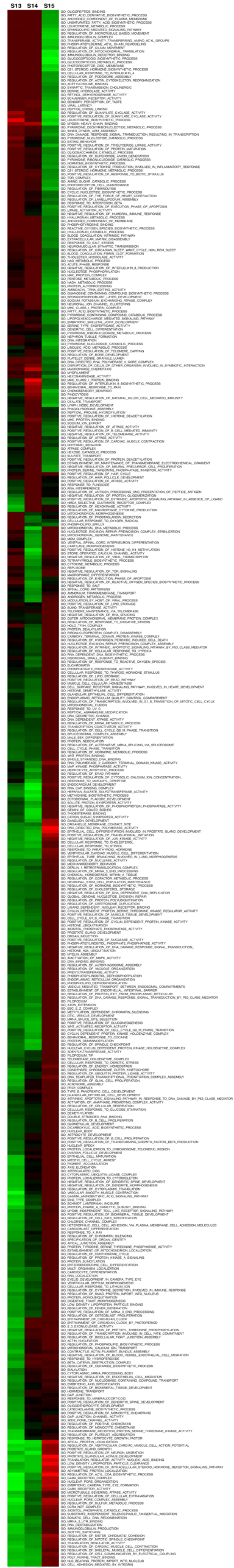

Supplement: S4 Fig — (TIFF) [file pone.0191452.s005.tiff]

## Enriched Pathways

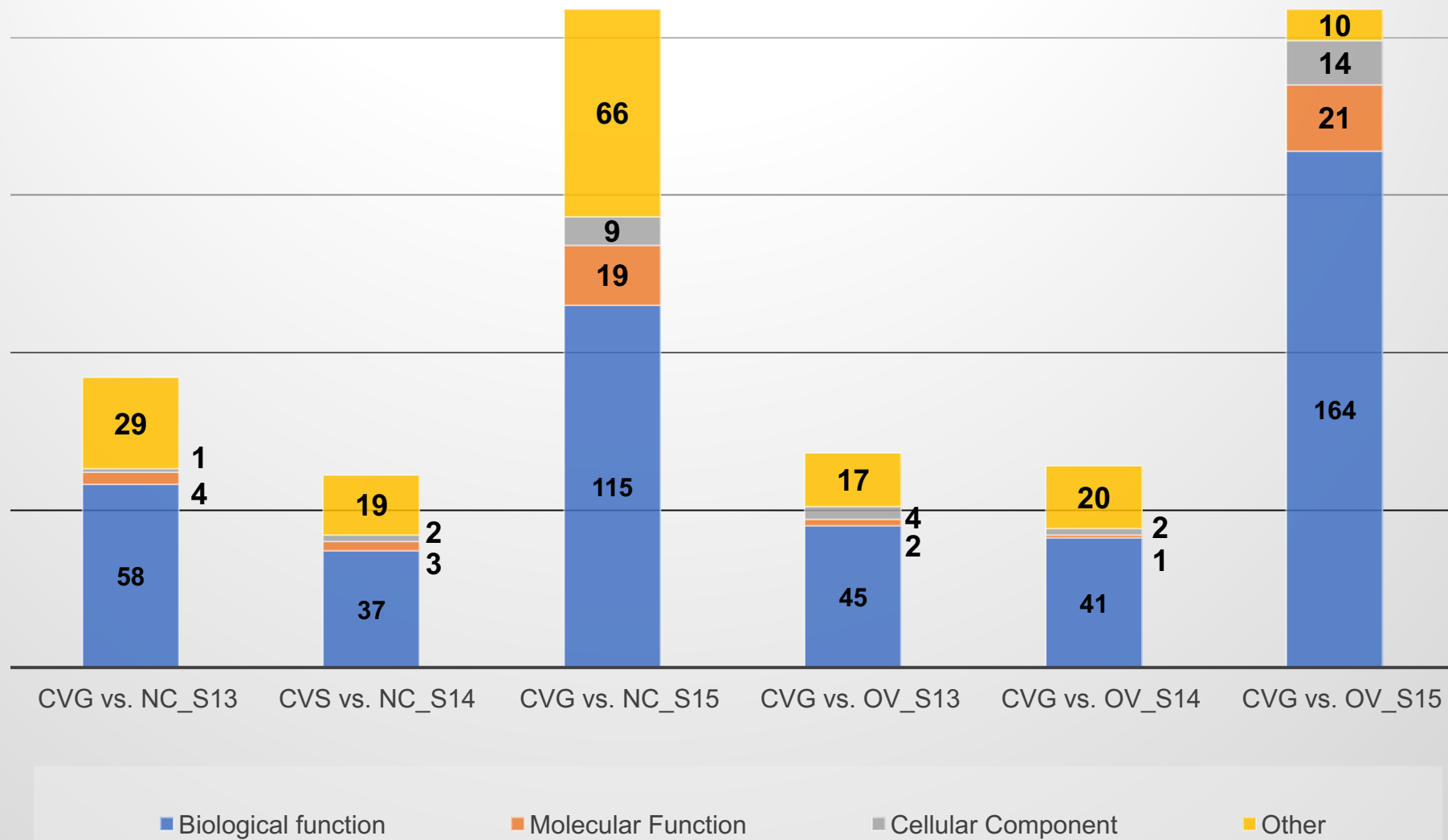

Supplement: S5 Fig — Comparisons among three tissue types at three time points. Blue bar: biological function; orange bar: molecular function; gray bar: cellular component; and yellow bar: others. The number of pathway is shown inside or next to each bar. S: Stage. (PDF) [file pone.0191452.s006.pdf]
