# Supplementary material for: Developmental profiling of microRNAs in the human embryonic inner ear
Source: PLoS One. 2018 Jan 26;13(1):e0191452. doi: 10.1371/journal.pone.0191452 (PMC5786302; doi:10.1371/journal.pone.0191452)
Supplement: S6 Fig — REVIGO treemap summarizing Gene Ontology biological process categories over-represented in CVG cells compared to NC cells at stage 14. All terms are included with a FDR adjusted p-value cutoff at 0.05 from the enrichment analysis. The relevance similarity C-score (uniqueness) cut-off is chosen at 0.7. The size of each rectangle is proportional to the uniqueness for that category. Red circles indicate relevant neuronal development pathways. (PDF) [file pone.0191452.s007.pdf]

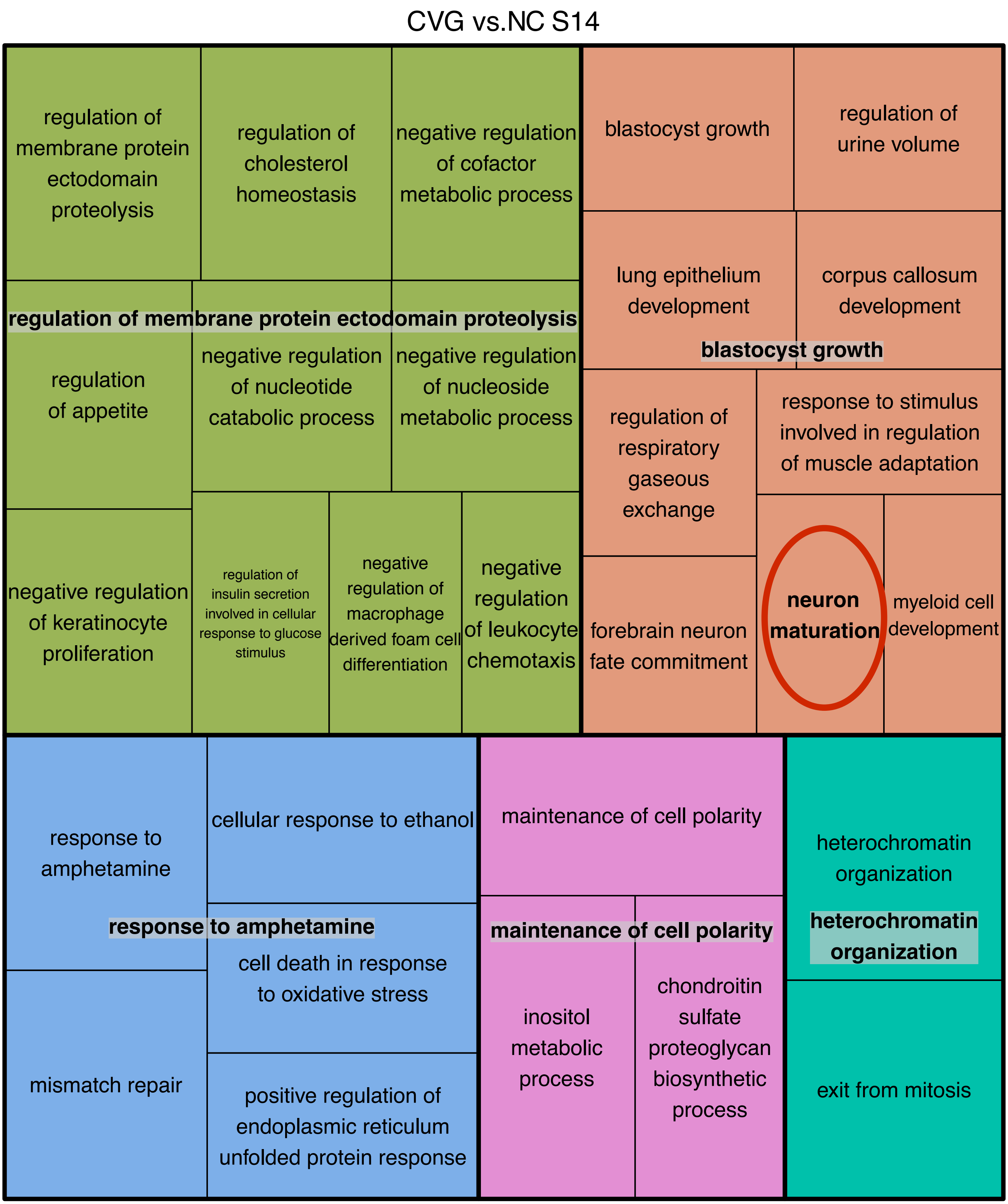
CVG vs.NC S14
regulation of membrane protein ectodomain proteolysis
regulation of cholesterol homeostasis
negative regulation of cofactor metabolic process
regulation of membrane protein ectodomain proteolysis
regulation of appetite
negative regulation of nucleotide catabolic process
negative regulation of nucleoside metabolic process
negative regulation of keratinocyte proliferation
regulation of insulin secretion involved in cellular response to glucose stimulus
negative regulation of macrophage derived foam cell differentiation
negative regulation of leukocyte chemotaxis
blastocyst growth
regulation of urine volume
lung epithelium development
corpus callosum development
blastocyst growth
regulation of respiratory gaseous exchange
response to stimulus involved in regulation of muscle adaptation
forebrain neuron fate commitment
neuron maturation
myeloid cell development
response to amphetamine
cellular response to ethanol
response to amphetamine
cell death in response to oxidative stress
mismatch repair
positive regulation of endoplasmic reticulum unfolded protein response
maintenance of cell polarity
maintenance of cell polarity
inositol metabolic process
chondroitin sulfate proteoglycan biosynthetic process
heterochromatin organization
heterochromatin organization
exit from mitosis
