# Supplementary material for: Developmental profiling of microRNAs in the human embryonic inner ear
Source: PLoS One. 2018 Jan 26;13(1):e0191452. doi: 10.1371/journal.pone.0191452 (PMC5786302; doi:10.1371/journal.pone.0191452)
Supplement: S1 File — (DOCX) [file pone.0191452.s015.docx]

**Gene set enrichment analysis**

The predicted miRNA targets were not filtered other than by species, and thus a limitation of this analysis is a potential increase in false positive results due to inaccurate target prediction. It is our opinion that, for an exploratory analysis, it is preferable to obtain some false positives rather than accept false negative results by overfiltering data. Additionally, filtering the target list could present a source of bias in the experiment. Validated miRNA targets are most likely related to the most popularly researched areas, such as cancer, leading to a significant knowledge bias [1]. TargetScan predicts targets based entirely on sequence similarity, and thus presents an unbiased source for target prediction at the miRNA to gene level.

Integrated gene set analysis was performed following the method of Garcia-Garcia et al. [2], incorporating the paradigm shift proposed by Godard and Eyall to prevent the influence of knowledge bias [1]. Starting with differential expression data, an $r$ statistic was computed for each tissue comparison across time points as

𝑟=-𝑠𝑖𝑔𝑛$\left( fold change \right)* log (P-value)$

The index thus contains information on the strength of differential expression (*P*-value) and the direction of the differential expression (the sign of the fold change). Although somewhat counterintuitive, it has been demonstrated that performing gene set analysis at the miRNA level by linking pathway information to miRNA reduces the effects of biased database information as compared to linking experimental miRNA differential expression results to their gene targets and analyzing at the gene level [1].

**References**

1. Godard P, Eyll J Van. Pathway analysis from lists of microRNAs : common pitfalls and alternative strategy. Nucleic Acids Res. 2015; 1–8. doi:10.1093/nar/gkv249

2. Garcia-Garcia F, Panadero J, Dopazo J, Montaner D. Integrated gene set analysis for microRNA studies. Bioinformatics. 2016;32: 2809–2816. doi:10.1093/bioinformatics/btw334
